# Temporal Activity and Co‐Occurrence Patterns of Sympatric Wild Ungulates in Baotianman, China

**DOI:** 10.1002/ece3.73897

**Published:** 2026-06-30

**Authors:** Zhaohui Xie, Song Yao, Liangfu Chen, Tongzhou Wang, Tong Liu, Yang Zheng, Liwen Zhu, Guangyi Lu

**Affiliations:** ^1^ School of Life Science and Bioengineering Henan University of Urban Construction Pingdingshan China; ^2^ Neixiang Management Bureau of Baotianman National Nature Reserve Neixiang China

**Keywords:** camera‐traps, co‐occurrence patterns, daily rhythm, ecological niches, ungulates

## Abstract

Species' habitat utilization reflects their habitat preferences and activity patterns. Understanding the coexistence mechanism of wild ungulates is critical for deciphering intra‐ and inter‐ species survival strategies. The interactions between species including predation, competition, symbiosis, and reproduction, are dynamic processes influenced by seasonal shifts, diel cycles, and weather variations. The Baotianman National Nature Reserve in northern China hosts diverse wild ungulate populations, yet their daily activity rhythms remain inadequately investigated. Leveraging camera‐trap data, we investigated the seasonal daily activity patterns of five sympatric wild ungulates (i.e., forest musk deer *Moschus berezovskii* , Siberian roe deer *Capreolus pygargus,* Reeve's muntjac *Muntiacus reevesi* , wild boar *Sus scrofa,* Chinese goral *Naemorhedus griseus* ), to assess temporal niche differentiation as a key coexistence mechanism. Comparative analyses revealed statistically significant seasonal differentiation in daily rhythms among these species. By Watson's *U* ^2^ test, the daily activity patterns differed significantly across forest musk deer, Reeve's muntjac, wild boar, and Chinese goral (*p* < 0.05). In contrast, the activity rhythms showed no significant differentiation between forest musk deer and Siberian roe deer. Notably, we found that forest musk deer, Siberian roe deer, and Reeve's muntjac were crepuscular, whereas wild boar and Chinese goral were diurnal. The highest degree of overlap coefficients was observed between the forest musk deer and the Siberian roe deer, with no significant difference in their diel activity rhythms. This study offers novel insights for developing conservation strategies for wild ungulates, and is crucial for maintaining ecological balance and biodiversity.

## Graphical abstract

Leveraging camera‐trap data, we investigated the seasonal daily activity patterns of five sympatric wild ungulates to assess temporal niche differentiation as a key coexistence mechanism. Comparative analyses revealed significant seasonal differentiation in daily rhythms among these species. By Watson's *U* ^2^ test, the daily activity patterns differed significantly across forest musk deer, Reeve's muntjac, wild boar, and Chinese goral (*p* < 0.05). We found that forest musk deer, Siberian roe deer, and Reeve's muntjac were crepuscular, whereas wild boar and Chinese goral were diurnal. The highest degree of overlap coefficients was observed between the forest musk deer and the Siberian roe deer (Δ = 0.90), with no significant difference in their diel activity rhythms (*U* ^2^ = 0.12, *p* > 0.01).

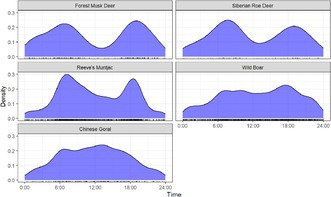

## 1 Introduction

The utilization of habitats by species reflects their preferences and activity patterns toward environmental factors (Chesson 2000; Wang et al. 2024). Interactions and influences among sympatric wild animal populations of different co‐occurring species operate on fine scales, and these dynamics directly shape species' behavioral patterns and resource utilization strategies (Franchini et al. 2023; Raimondi et al. 2023). Such interactions encompass diverse forms, including predation, competition, symbiosis, and reproduction, with their occurrence and variability closely tied to seasonal shifts, daily cycles, and weather fluctuations (Ikeda et al. 2016; Noor et al. 2017; Li et al. 2021). Investigating fine‐scale interactions provides critical insights into ecosystem complexity, facilitating the development of predictive models for ecological dynamics and targeted conservation strategies (Bu et al. 2016). Understanding the daily activity rhythms of different ungulate species can facilitate interspecific coexistence and enhance adaptive capacity, promote the efficient utilization of natural resources, and is of great significance for sustaining individual survival and population reproduction (Finke and Snyder 2008). Ungulates' activity rhythms are also shaped by interspecific competition and niche differentiation. Interspecific competition can induce overlapping activity rhythms among ungulates occupying the same niche (Gao et al. 2020; Bhasin et al. 2024; Raimondi et al. 2023), thereby mitigating resource competition pressure. Ecological niche differentiation, by contrast, spatiotemporally disperses the activity patterns of ungulates, further alleviating interspecific competitive stress (Darmon et al. 2012; Raimondi et al. 2023). Considerable variation in activity rhythms exists across ungulate species, which serves as a temporal niche partitioning mechanism enabling their peaceful coexistence within shared habitats. However, over recent decades, anthropogenic disturbances have imposed increasingly intense pressures on wildlife ecosystems (Gaynor et al. 2019; Orrick et al. 2024). The expansion and development of human societies have altered the habitats and activity schedules of wildlife, and modified the interaction dynamics between predators and prey (Gao and Susan 2023). Therefore, those current research topic of protecting wildlife and their ecological sustainability from human activities is particularly crucial.

Habitat selection and interspecific competition are key drivers of species abundance and distribution at the local scale (Hysen et al. 2025). The habitat and interspecific relationships of rare species require special attention, as biodiversity losses often occurs primarily through the disappearance of these species from regional assemblages (Guan et al. 2009). The forest musk deer ( *Moschus berezovskii* Flerov, 1929), a solitary ruminant endemic to southwestern China, is one of the five extant musk deer species globally. This species inhabits high‐altitude closed‐canopy coniferous and broad‐leaved mixed forests as a specialized ungulate. Due to its restricted distribution range and small population size (Ohtaishi and Gao 1990), it has been classified as a National First‐grade Protected Wild Animal in China and categorized as Endangered on the IUCN Red List of Threatened Species (Wang and Harris 2015). Forest musk deer, and Reeves's muntjac ( *Muntiacus reevesi* Ogilby, 1839) have similar habitat (Chen et al. 2024). According to the IUCN Red List of Threatened Species, both the Siberian roe deer ( *Capreolus pygargus* Pallas, 1771) and Reeves's muntjac are currently assessed as “Least Concern” (Lovari et al. 2016; Timmins and Chan 2016). In contrast, the Chinese goral ( *Naemorhedus griseus* Milne‐Edwards, 1871) is assessed as “Vulnerable” (Duckworth et al. 2008). The Siberian roe deer has a broad distribution across continental Asia and Eastern Europe. Its range extends westward from the Khoper River and Don River bend, eastward across the Ural Mountains and southern Siberia through northern Mongolia, and reaches its easternmost limit along coastlines regions of the East Sea, and the Yellow Sea basin, including the Korean Peninsula (Feng et al. 2021). Reeves's muntjac, a species of Muntiacus (Cervidae), is an endemic mammal of China. As the smallest muntjac, it is distributed across Mid‐Eastern China (Smith and Xie 2013). The Chinese goral is a small goat‐like animal (Ariyaraphong et al. 2021; Wu et al. 2024). Its distribution spans northern, central, southern and eastern China, with extensions to northeastern India, western Myanmar, northeastern Thailand. In China, it is classified as a National Second‐Class Protected Wild Animal and is listed in the CITES appendix I of the Convention on International Trade in Endangered Species of Wild Fauna and Flora (CITES 2021; www.cites.org). Additionally, the forest musk deer, Reeves's muntjac, Chinese goral, and Siberian roe deer are recorded in the Red List of Biodiversity in China: Vertebrate Volume 2020 (www.mee.gov.cn) with detailed assessment of their conservation status. The wild boar ( *Sus scrofa* Linnaeus, 1758) are the most widespread wild ungulate globally (Horčičková et al. 2019; Sales et al. 2017). Its current distribution covers all continents except Antarctica, rendering it one of the most widespread terrestrial mammals (Lewis et al. 2017). Renowned for their exceptional adaptability, wild boar exhibit the highest reproductive rate among ungulates, a key factor underpinning to their ecological success (Massei et al. 1997; Fonseca et al. 2011) classified as an invasive species in numerous regions, they pose significant threats, including agricultural crop damage, livestock disease transmission, vehicular collisions, and adverse impacts on native ecosystems (Guo et al. 2017).

**FIGURE 1:**
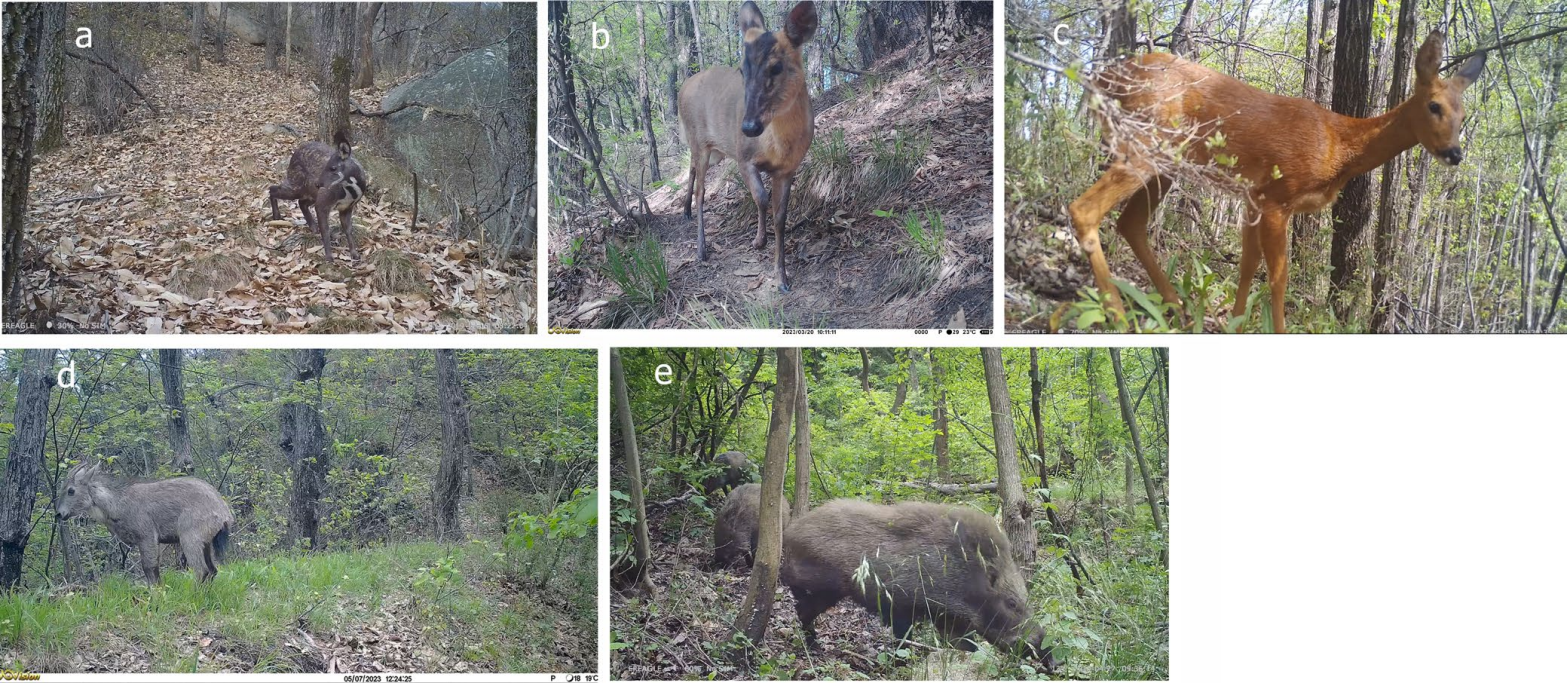
Five sympatric wild ungulate species in Baotianman National Nature Reserve, Henan, China. (a) Forest musk deer *Moschus berezovskii* , (b) Reeve's muntjac *Muntiacus reevesi*, (c) Siberian roe deer *Capreolus pygargus* , (d) Chinese goral *Naemorhedus griseus* , (e) Wild boar *Sus scrofa* .

Infrared camera technology has become a widely adopted tool for monitoring and investigating of mammalian species resources in nature reserves worldwide (Zhang et al. 2014; Craig et al. 2022). This methodology offers distinct advantages in studying animal behavior rhythms. As it enables continuous and unrestricted data collection, reduces interference with animals, geographical constraints and limitations in field personnel availability, thereby ensures the data objectivity (Forrester et al. 2016; Ahumada et al. 2019). The activity rhythm of animals refers to species‐specific temporal patterns of behavioral intensity across diel and seasonal cycles, reflecting adaptive responses to environmental and ecological variables (Li et al. 2014; Xiao et al. 2022; Proudman and Allen 2026). These studies are crucial for understanding the behavioral patterns of animals and provide a scientific basis for formulating wildlife protection strategies, thereby supporting the implementation of more effective management and conservation measures (Xiao et al. 2023). By analyzing the daily activity rhythms of these ungulates, it is possible to better predict their needs to protect their habitats and intervene when necessary to reduce the adverse effects of human activities. Based on infrared camera data from April 2019 to December 2023 in Baotianman National Nature Reserve, this study analyzed five ungulates, including forest musk deer, Siberian roe deer, Reeve's muntjac, wild boar, and Chinese goral (Figure 1). We employed R software (Version 4.0.1) to analyze the activity rhythms of these animals (R Core Team 2025), which facilitates understanding the fine‐scale interactions among ungulate species in the study area. We hypothesized that these sympatric ungulates exhibit temporal niche differentiation to alleviate interspecific competition, and that their activity rhythms differ between warm and cold seasons. Specifically, we predicted that species with similar ecological characteristics would have higher temporal activity overlap, whereas the dominant species (wild boar) would exhibit more flexible activity patterns. Understanding how ungulates schedule their daily activities is key to grasping their ecological requirements and potential responses to external disturbances, and such analysis can provide a scientific foundation for developing effective conservation strategies and management practices for those species.

## 2 Materials and Methods

### 2.1 Study Area

**FIGURE 2:**
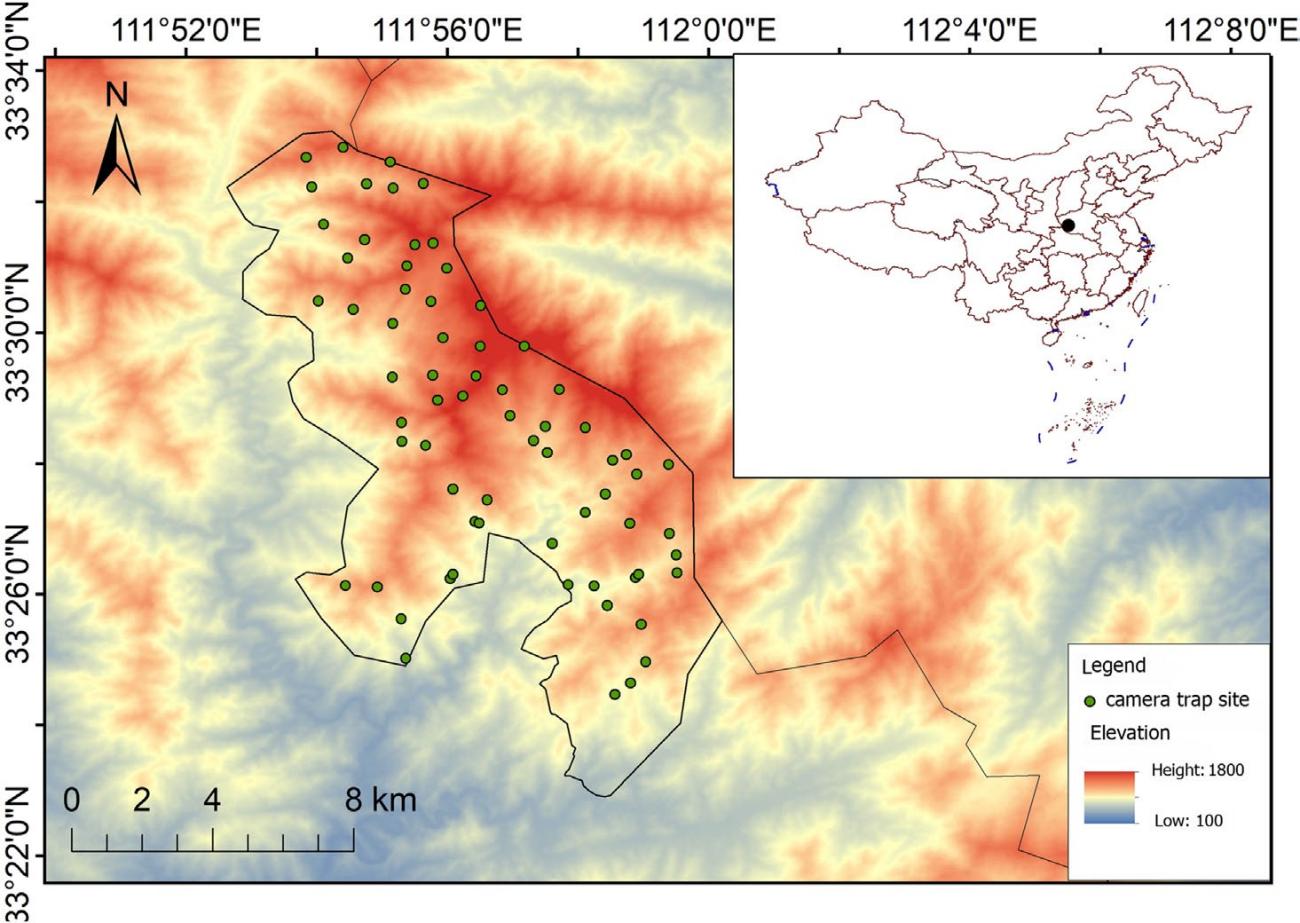
Distribution of camera trapping sites across Baotianman National Nature Reserve (33°25′–33°36′ N, 111°53′–112°04′ E), Henan, China. Elevations ranging from approximately 720 m to 1750 m. A total of 63 camera traps for wild ungulate surveys. Green dots denote camera trap locations.

Baotianman National Nature Reserve (33°25 ~ 33°36′ N, 111°53′ ~ 112°04′ E) is located in Henan Province, China (Figure 2). Covering an area of approximately 9304 km^2^, the reserve spans an elevation range from 500 m to 1830 m. It has an average annual temperature of 15.1°C. The coldest month is January, with an average temperature of 1.5°C and an extreme minimum temperature of −14.8°C, while the hottest month is July, with an average of 27.8°C and reaching an extreme maximum of 41.2°C. The summer hot season lasts for 81.1 days, and the winter cold season persists for 73.6 days. The annual average precipitation is 886 mm (Ye and Liu 2017). The reserve is dominated by warm temperate deciduous broad‐leaved forests and mixed coniferous‐broadleaf forests, forming a typical forest ecosystem. As the cornerstone of regional ecological balance, the reserve's comprehensive transitional forest ecosystem provides critical habitats for rare wild animals and plants, plays a pivotal role in climate regulation, water conservation, and other ecological processes, and is highly difficult to restore once degraded. Additionally, scattered evergreen forest patches are distributed in low‐lying valley areas, composed of plant species such as *Abies chensiensis* Tiegh., *Corylus chinensis* Franch., *Picea neoveitchii* Mast., *Cercidiphyllum japonicum* Sieb. & Zucc., *Emmenopterys henryi* Oliv., *Eucommia ulmoides* Oliv., etc. These species enhance the seasonal dynamics and biodiversity of the reserve. Situated at the ecotone between the northern subtropical and warm temperate biogeographical zones, the region exhibits typical East Asian monsoon transitional climate characteristics, which create a diverse array of microhabitats and support high species richness (Yao et al. 2012; Ye and Liu 2017).

### 2.2 Data Collection

We conducted infrared camera‐trapping from March 2019 to December 2023 in Baotianman Nature Reserve, with placement prioritizing animal trails, foraging areas, water sources—habitats and spots frequently used by target species. We set up 63 camera traps (Bestguarder SG‐990 V, Nighthawk4, Shenzhen Siyuan Digital Technology Co. Ltd., Shenzhen, China) at different sites above sea level from 720 to 1750 m to survey wild ungulates. Generally, we fixed cameras on the trees at a height of 20–150 cm, facing animal tracks, water sources, natural salt lick sites, or resting sites. Camera sites were spaced at least 300 m apart from each other. Additionally, no baits were used in camera sites to capture the natural wildlife behavior (Raimondi et al. 2023). According to the unified survey plan of the biodiversity observation network, Baotianman National Nature Reserve and its surrounding areas were divided into 2 km × 2 km grids using ArcGIS 10.2. When planning observation sites, we conducted a comprehensive assessment to evaluate the vegetation types, elevation, anthropogenic environmental impacts, and wildlife distribution patterns of each grid unit. Finally, areas composed of 20 adjacent grids were selected as the geographic positioning reference for setting up infrared cameras. Then, we placed 63 infrared cameras within the target grid. During the investigation process, the researchers regularly collected data and inspected the infrared camera, replaced the storage card, and adjusted the camera's position appropriately. During the investigation period, the infrared camera was generally inspected and maintained every 3–6 months (Zhang et al. 2023). By ensuring the distribution of cameras in this way, we took into account not only ecological diversity, but also hotspots of human and animal activities, thus enabling more effective wildlife population monitoring.

### 2.3 Data Analysis

At each camera placement site station, photographic and video data were systematically categorized by geographic location, hourly interval, and date. To ensure statistical independence of wildlife capture events, consecutive photograph records of conspecific individuals captured within 30‐min intervals were consolidated into a single occurrence event, and for all subsequent analyses, only independent detections were included (Li et al. 2010, 2020; Caravaggi et al. 2017). The number of effective camera trap days was calculated following standard protocols (O'Connell et al. 2011). For each camera, the effective survey days were defined as the total duration from the date of successful installation to either the date when the camera ceased operation (e.g., battery failure, malfunction, or memory card full) or the date of retrieval at the end of the study period. In cases where the exact cessation date could not be directly verified from camera logs or maintenance records—such as when cameras were lost or damaged—we conservatively estimated the end date as the date of the last captured photograph or video as a surrogate (O'Connell et al. 2011). The total effective camera trap days for the study were then calculated as the sum of effective days across all cameras. Since the recorded clock time of species detections lacks inherent biological significance, we converted the clock time of independent events to solar time using the sunrise and sunset time each day at the coordinates of the center of the reserve (Sogliani et al. 2025). All the solar times were treated as part of a 24‐h cycle.

Researchers performed a quantitative analysis of the diel activity patterns of the five ungulate species using the “overlap” package in R (Meredith and Ridout 2024). To determine whether each species exhibited random activity patterns (Viviano et al. 2021), the study further employed the HR_test function in the CircMLE package to conduct the Herman‐Larson test (Fitak and Johnsen 2017). Test results indicated that all species except the Chinese goral exhibited non‐random diel activity patterns (*p* < 0.001). This finding indicates that the activity rhythms of the other four species (forest musk deer, Siberian roe deer, Reeves's muntjac, and wild boar) are not randomly generated but are jointly regulated by intrinsic physiological requirements (such as the inherent cycles of foraging, reproduction, and rest) and environmental factors (such as temperature, light availability, predator activity, and resource distribution). Conversely, the random diel activity pattern observed in the Chinese goral (*p* = 0.215) may be linked to anthropogenic disturbances.

We compiled independent detection events for five wild ungulates species, with the time and date recorded based on the first photograph of each independent event. To assess interspecific differences in diel activity patterns, we generated a null distribution of temporal overlap indices by resampling with replacement from the pooled data set. This study employed Watson's Two‐Sample *U* ^2^ Test to assess the significance of activity pattern differences, both between species and between the cold seasons (i.e., October to March) and warm seasons (i.e., April to September) within each species, with a significance level set at *p* = 0.05 (Zar 2010). To further examine seasonal variations in temporal niche overlap, we also classified the study period into four meteorological seasons based on local climatic characteristics: spring (March–May), summer (June–August), autumn (September–November), and winter (December–February).

Relative abundance index (RAI) was calculated based on independent valid photographs using the formula: RAI = (Ai/N) × 100 where Ai denotes the number of independent valid photos of species *i* (*i* = 1,2,3,… corresponding to the five ungulate species), and *N* represents the total number of independent valid photographs. RAI is expressed as a percentage, reflecting the relative abundance of each species in the study area.

We calculated temporal niche overlap coefficients (Δ) for the five ungulate species across four distinct seasons. The Δ metric quantifies the degree of temporal niche overlap between species, with values ranging from 0 to 1: Δ value of 0 indicates no overlap, while a value of 1 indicates complete overlap.

Following the classification criteria established from previous studies (Monterroso et al. 2014; Sogliani et al. 2021), the degree of temporal niche overlap between species was categorized into four levels: “very low overlap” (Δ ≤ 0.35), “low overlap” (0.35 < Δ ≤ 0.50), “moderate overlap” (0.5 < Δ ≤ 0.75), and “high overlap” (Δ > 0.75). To evaluate the statistical reliability of the overlap coefficients, we employed the bootstrap resampling method (10,000 replicates; Meredith and Ridout 2024) to estimate the 95% confidence intervals (hereafter referred to as 95% CI) of the Δ for each species of pair. Since the temporal overlap coefficient is solely a descriptive statistic and does not have an associated significance test for pairwise comparisons (Monterroso et al. 2014), we further conducted the Mardia‐Watson‐Wheeler test (W‐) using the “circular” package in R (Mori et al. 2021) to statistically assess whether significant differences in diel activity times existed between pairwise combinations of five ungulate species: forest musk deer, Siberian roe deer, Reeve's muntjac, wild boar, and Chinese goral.

To accurately analyze biologically meaningful activity patterns, we converted the recorded clock times of all independent detection events to true solar time, calculated specifically for the geographic coordinates of each camera trap location. We used Package suncalc (Version 0.5.0) in R (Version 3.6.1) to estimate time of complete darkness and sunrise on the study site. We derived precise sunrise and sunset times for the date and location of each observation. We then calculated the hourly time difference between each animal detection and these daily solar events. Based on these solar‐referenced intervals, we categorized each observation into one of four distinct temporal phases: “around sunrise” (1 h post‐sunrise to 1 h pre‐sunrise), “around sunset” (1 h pre‐sunset to 1 h post‐sunset), “daytime” (1 h post‐sunrise to 1 h pre‐sunset), and “nighttime” (1 h post‐sunset to 1 h pre‐sunrise). This operational definition for classifying diel activity phases is well‐established in wildlife ecology (Rowcliffe et al. 2015; Proudman and Allen 2026).

Diel phenotype classification: Based on the solar‐defined intervals above, We adopted the analytical framework developed by Gerber et al. (2024), a model‐based hypothesis framework implemented in the Diel.Niche R package, to quantify diel activity phenotypes of focal species via its online Shiny platform (https://shiny.uri.edu/bgerber/DielNiche/). Analyses were run under the General hypothesis set, which assesses the statistical support for standard diel phenotypes based on species' utilization of each diel period and distinguishes bimodal from trimodal activity modalities. The raw data and code used in this study can be found in Table S1 and Code.docx.

## 3 Results

Over the study period, 63 camera trap sites amassed 155,011 trap‐days, resulting 4377 independent ungulates detection. Species‐specific detection counts were as follows: forest musk deer 351 (8.0%), Siberian roe deer 124 (2.8%), Reeve's muntjac 1584 (36.1%), wild boar 1923 (43.9%), and Chinese goral 395 (9.0%).

### 3.1 Single‐Species Seasonal Relative Activity Intensity

**FIGURE 3:**
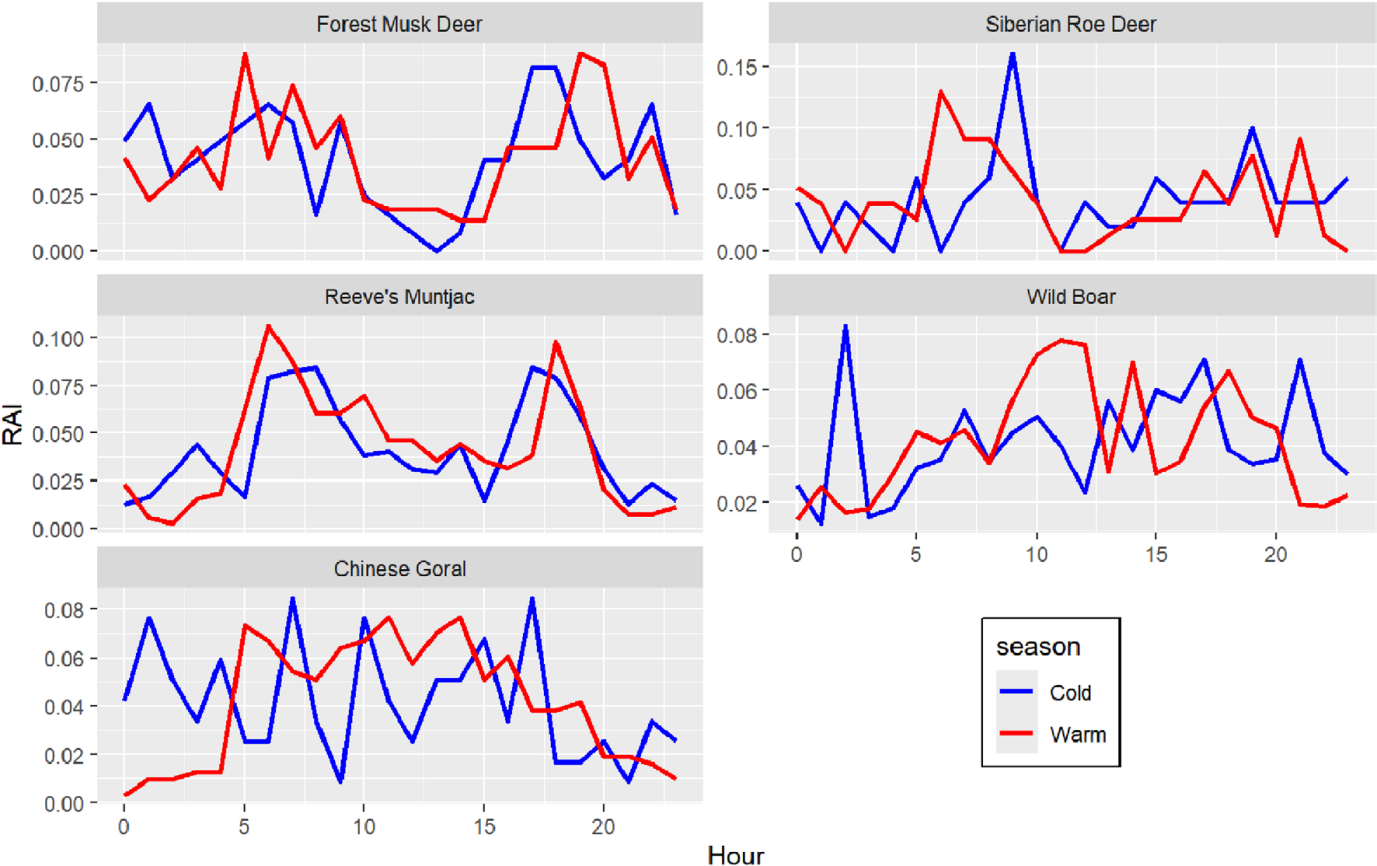
Distribution of camera trapping sites across Baotianman National Nature Reserve (33°25′–33°36′ N, 111°53′–112°04′ E), Henan, China. Elevations ranging from approximately 720 m to 1750 m. A total of 63 camera traps for wild ungulate surveys. Green dots denote camera trap locations.

The seasonal relative activity intensity (warm vs. cold season) of the five ungulate species is presented in the Figure 3. Forest musk deer and Reeves's muntjac exhibited higher relative daily activity intensity during twilight hours in cold and warm seasons, while notably lower intensity during other periods. The Siberian roe deer displayed elevated activity levels between 5:00 and 10:00 (24‐h clock) in both seasons, with reduced intensity during the remainder of the diel cycle. Wild boar maintained relatively stable daily activity levels throughout the diel cycle across both seasons. For the Chinese goral, warm‐season activity intensity was noticeably higher during daytime, whereas in the cold season, activity exhibited more irregularly fluctuations—a pattern potentially driven by temperature variations.

### 3.2 Seasonal Daily Activity Patterns

**FIGURE 4:**
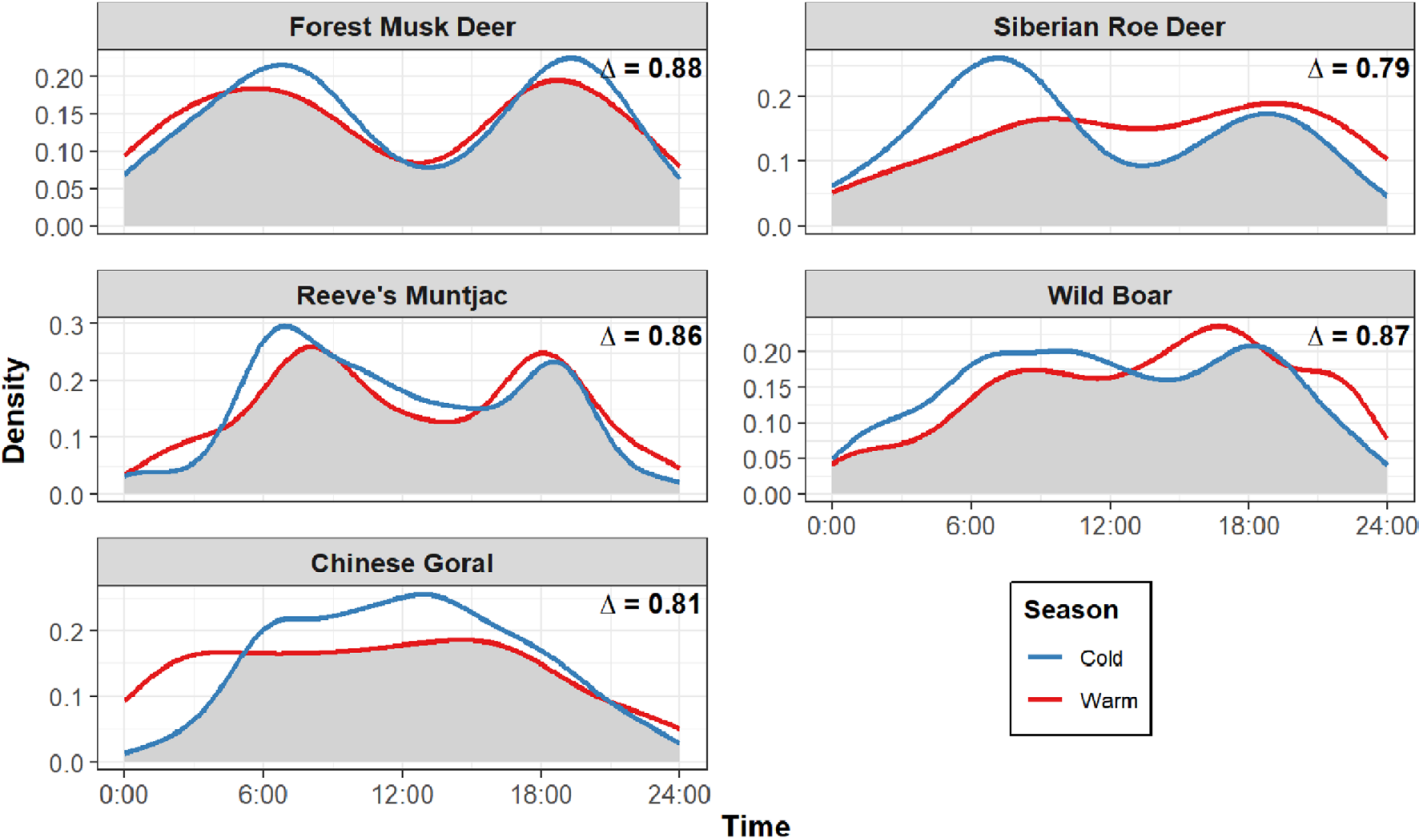
The seasonal daily activity patterns of five ungulate species in Baotianman National Nature Reserve. Blue lines denote the cold season, and red lines denote the warm season. The y‐axis is the “Kernel Density Estimates”.

The seasonal daily activity patterns of five ungulate species (Figure 4) and their statistical significance (Table 1): forest musk deer and Reeves's muntjac exhibited crepuscular activity patterns. During the warm season, both species displayed two distinct activity peaks (dawn and dusk). In the cold season, however, the crepuscular activity intensity of the forest musk deer diminished, with its peak activity shifting earlier. While Reeves's muntjac also showed reduced crepuscular activity with a noticeable shift toward later times.

**TABLE 1:** The seasonal daily activity patterns of five sympatric wild ungulates in Baotianman, China during March 2019 to December 2023. Overlap coefficients of species in the cold and warm seasons.

Species	Overlap coefficient	CI	*p*	*U* ^2^
Forest musk deer	0.88	0.77–0.91	0.26	0.06
Siberian roe deer	0.80	0.60–0.83	0.096	0.15
Reeve's muntjac	0.86	0.81–0.88	< 0.01	0.29
Wild boar	0.87	0.83–0.90	< 0.01	0.48
Chinese goral	0.81	0.73–0.86	< 0.01	0.32

The Siberian roe deer was primarily diurnal and crepuscular. It displayed a clear bimodal pattern in the warm season, with peaks following sunrise and preceding sunset. Although the activity peaks were less pronounced in the cold season, their timing shifted later compared to the warm season. Wild boar exhibited diel activity throughout the 24‐h cycle, with higher intensity during daytime than nighttime. Compared to the cold season, the species increased pre‐sunrise activity during the warm season while reducing post‐sunset activity. The Chinese goral was mainly diurnal. Its peak activity occurred around noon in the warm season, shifting to the afternoon during the cold season.

### 3.3 Annual Scale Daily Activity Rhythms

**FIGURE 5:**
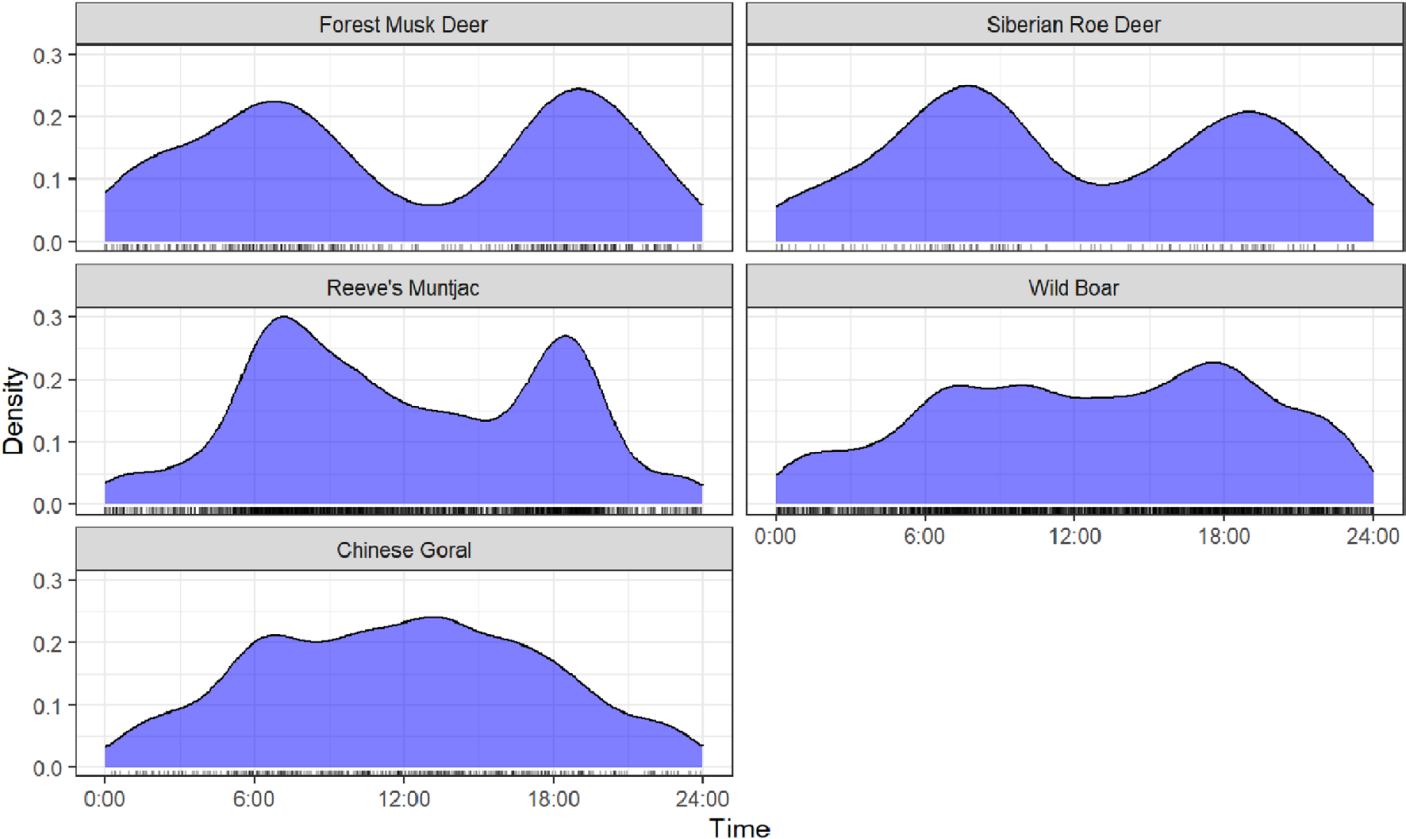
Annual scale daily activity rhythms of five ungulate species in Baotianman National Nature Reserve. The y‐axis is the “Kernel Density Estimates”.

The annual‐scale daily activity patterns of five ungulate species in Baotianman National Nature Reserve are shown in Figure 5. The Chinese goral displayed distinct diurnal activity, with a prominent activity peak between 12:00 and 15:00. The forest musk deer, Reeves's muntjac, and Siberian roe deer all exhibited clear bimodal activity patterns, characterized by elevated activity during crepuscular hours (dawn and dusk). This behavioral strategy likely enables them to avoid midday heat and reduce predation risk. Notably, the wild boar population was significantly larger than those of the Chinese goral, forest musk deer, Reeves's muntjac, and Siberian roe deer. Its activity pattern was more clearly compared to the other four species: a morning activity peak occurred between 7:00 and 10:00, followed by a decline in activity from 10:00 to 15:00. Activity intensity increased again after 15:00, reaching the highest peak at approximately 18:00, and then gradually decreased thereafter. Despite the reduced activity frequency post‐18:00, the overall activity level of the wild boar population remained relatively high throughout the nighttime.

### 3.4 Temporal Niche Overlap Among Species

**FIGURE 6:**
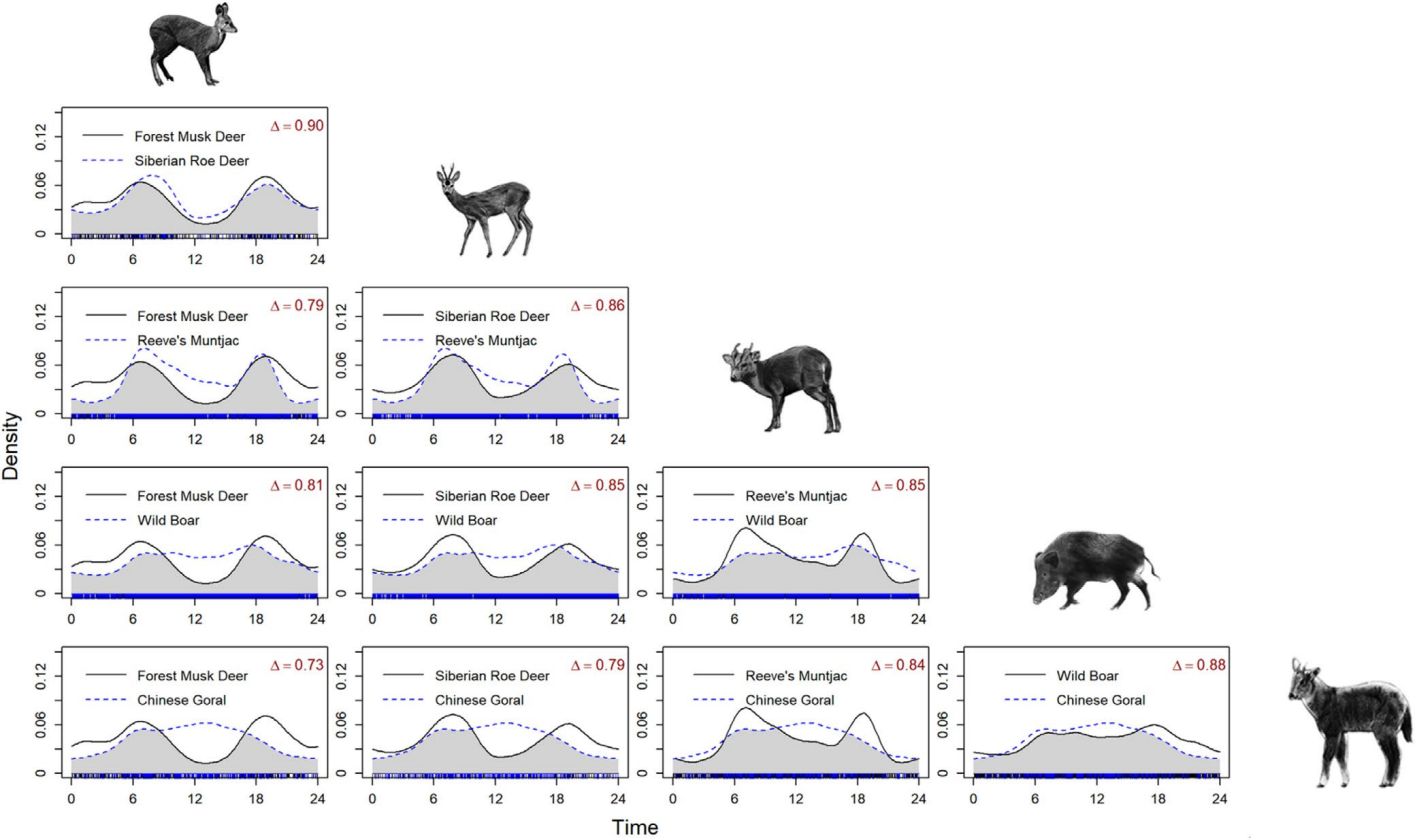
Daily activity patterns and temporal overlapped different pairs of five wild ungulate species. Overlaps are represented by the shaded gray area. The y‐axis is the “Kernel Density Estimates”. Overlap coefficients (∆) are shown in brackets.

To investigate temporal niche partitioning among five ungulate species, we calculated pairwise temporal niche overlap coefficients using data collected from March 2019 to December 2023 (Figure 6 and Table 2).

**TABLE 2:** Daily activity patterns of five sympatric wild ungulates were analyzed using data collected from March 2019 to December 2023 at Baotianman, China, and pairwise temporal niche overlap coefficients among these species.

Species pairs	Overlap coefficient	CI	*p*	*U* ^2^
Forest musk deer, Siberian roe deer	0.90	0.79–0.92	0.46	0.12
Forest musk deer, Reeve's muntjac	0.79	0.73–0.83	< 0.01	1.99
Forest musk deer, Wild boar	0.81	0.76–0.83	< 0.01	1.49
Forest musk deer, Chinese goral	0.73	0.67–0.77	< 0.01	1.92
Siberian roe deer, Reeve's muntjac	0.86	0.77–0.89	0.014	0.31
Siberian roe deer, Wild boar	0.85	0.76–0.89	< 0.01	0.31
Siberian roe deer, Chinese goral	0.79	0.69–0.83	< 0.01	0.54
Reeve's muntjac, Wild boar	0.85	0.82–0.88	< 0.01	1.09
Reeve's muntjac, Chinese goral	0.84	0.79–0.87	< 0.01	0.46
Wild boar, Chinese goral	0.88	0.83–0.91	< 0.01	0.53

The highest degree of overlap coefficients was observed between the forest musk deer and the Siberian roe deer (Δ = 0.90), with no significant difference in their diel activity rhythms (*U* ^2^ = 0.12, *p* > 0.01). Although the forest musk deer exhibited high overlap coefficients with Reeves's muntjac, wild boar, and Chinese goral (Δ > 0.75), their diel activity rhythms differed significantly (*p* < 0.01). Furthermore, the diel activity patterns of Siberian roe deer differ significantly from those of Reeves's muntjac, wild boar, and Chinese goral (*p* < 0.05). Significant differences (*p* < 0.01) were also detected between Reeves's muntjac and wild boar, Reeves's muntjac and the Chinese goral, as well as between the wild boar and the Chinese goral.

### 3.5 Analysis of Diel Activity Patterns

Examination of diel time budgets revealed distinct activity allocation patterns among the five ungulate species. Forest musk deer, Siberian roe deer, and Reeve's muntjac exhibited pronounced crepuscular activity peaks (Figure 5), characterized by clear bimodal patterns at dawn and dusk. However, despite these pronounced peaks, their overall activity was distributed throughout the day. Our model outputs provided strong statistical evidence that forest musk deer exhibit a diurnal–nocturnal activity phenotype. We obtained moderate support (model probability > 0.75) for a diurnal–nocturnal pattern in Siberian roe deer, Reeve's muntjac, and wild boar, whereas Chinese goral were classified as cathemeral (Table 3). Our findings partially contradict previous published results (Cox et al. 2021): prior work defined forest musk deer as strictly nocturnal, Siberian roe deer, Reeve's muntjac, and wild boar as cathemeral, and Chinese goral as crepuscular. This seeming contradiction arises from our definition of twilight as the narrow one‐hour windows before and after sunrise and sunset. While these short windows capture the most intense peaks of crepuscular activity, the activity during the broader dawn and dusk periods extends beyond these strict boundaries, falling into daytime and nighttime categories. These divergent diel activity patterns reflect their distinct ecological niches and adaptive strategies.

**TABLE 3:** Detection frequencies of five sympatric wild ungulates in Baotianman, China during March 2019 to December 2023, and posterior model probabilities of support from the general hypothesis set. Crep, Crepuscular; Diur, Diurnal; Noct, Nocturnal.

Species	Day	Twilight	Night	Diurnal	Nocturnal	Crepuscular	Cathemeral	Diur‐Crep	Diur‐Noct	Crep‐Noct
Forest musk deer	169	19	163	0.00	0.00	0.00	0.00	0.00	1.00	0.00
Siberian roe deer	55	11	58	0.00	0.00	0.00	0.14	0.00	0.86	0.00
Reeve's muntjac	695	159	730	0.00	0.00	0.00	0.25	0.00	0.75	0.00
Wild boar	646	189	1088	0.00	0.00	0.00	0.16	0.00	0.84	0.00
Chinese goral	157	45	193	0.00	0.00	0.00	0.58	0.00	0.42	0.00

## 4 Discussion

Investigating the activity patterns and time allocation of wildlife is essential for formulating science‐based conservation strategies for multiple species within protected areas. This study revealed the five wild ungulate species in Baotianman National Nature Reserve (forest musk deer, Siberian roe deer, Reeves's muntjac, wild boar, and Chinese goral) exhibited high pairwise temporal niche overlap (Δ > 0.75). Notably, the wild boar emerged as the dominant species, with a relative abundance significantly higher than that of the other four species. These findings suggest that the wild boar population was overly abundant and shared overlapping habitats with the coexisting ungulates. Therefore, it is recommended that targeted management measures be implemented to regulate the growth of the wild boar population within the reserve.

Since infrared cameras had a consistent detection probability for ungulates, the frequency of independent photographic records was used to infer their activity intensity (Wen et al. 2020). Furthermore, investigating daily activity rhythms is widely recognized as crucial for understanding animals' ecological behavioral strategies, as well as interspecific relationships (Linkie and Ridout 2011; Chen, Shu, and Xiao 2019). Our results indicate that the forest musk deer, Siberian roe deer, and Reeves's muntjac exhibited annual bimodal activity patterns, with their peak activities concentrated around dawn and dusk. In contrast, the wild boar displayed two less distinct activity peaks and maintained relatively stable activity throughout the day. The Chinese goral, however, showed a unimodal pattern, with its activity peak primarily occurring during daytime. This suggests potential temporal niche partitioning among these sympatric ungulate species.

As illustrated in Figure 4, both the forest musk deer and the Reeves's muntjac exhibited two distinct activity peaks in both warm and cold seasons, consistently occurring around dawn and dusk. This aligns with the findings of Wang et al. (2024). However, compared to the cold season, the dusk activity peaks for both species shifted later in the warm season. Notably, the dawn peak for forest musk deer also shifted later, whereas that of Reeves's muntjac shifted earlier. This indicates seasonal temporal niche differentiation between the two species. Siberian roe deer showed two clear activity peaks in the warm season, but these peaks became less distinct and shifted later in the cold season. These observation suggest that temperature is likely a key factor influencing their daily activity patterns, supporting the conclusions of Banjade et al. (2023). Collectively, these results highlight the critical role of temperature in driving seasonal shifts in the daily activity patterns of Siberian roe deer.

Previous studies have characterized the wild boar's activity pattern as cathemeral, lacking distinct activity peaks (de Assis et al. 2020), a finding corroborated by our data. Considering the seasonal variation in wild boar diets (Lee and Lee 2019), the observed seasonal variations in their daily activity rhythms are likely associated with fluctuations in food availability. For the Chinese goral, a clear unimodal pattern was observed during the warm season, whereas its activity in the cold season became more consistent and lower than the warm‐season peak. This pattern may be linked to energy requirements, ambient temperature, and adaptive survival strategies.

Temporal niche partitioning is likely an evolutionary strategy to reduce competition for limited resources. The five ungulate species exhibited moderate overlap in their daily activity patterns, with the highest overlap (Δ = 0.90) recorded between the forest musk deer and Siberian roe deer. Although both species are primarily crepuscular, their peak activity times within these periods differ significantly. This suggests that they achieved ecological separation and stable coexistence by utilizing food resources at different heights (Deng et al. 2020; Zhang 2014). Potential interspecific competition appeared more intense between Reeves's muntjac and the Chinese goral, as they shared similar diets niches (Hu et al. 2025; Tang et al. 2019) and exhibited highly overlapping activity patterns. The Reeves's muntjac displayed a typical bimodal pattern active at dawn and dusk, while the Chinese goral exhibits a unimodal pattern concentrated in the daytime. Thus, temporal niche differentiation likely mediates interspecific competition between these two species (Tian et al. 2020; Chen, Xiao, et al. 2019; Li et al. 2014). As the largest‐bodied and only omnivorous ungulate in Baotianman National Nature Reserve, wild boar occupies a dominant position in resource acquisition and habitat utilization (Wang et al. 2024). Activity rhythm analysis confirmed significant temporal niche partitioning between wild boars and other ungulates. This differentiation effectively alleviates interspecific competition and serves as a key mechanism promoting stable coexistence within the ungulate community (Lashley et al. 2018; Hearn et al. 2018; Karanth et al. 2017). However, the relatively high overlap coefficients (Δ > 0.8) between wild boars and other ungulates indicated potential competition for food resources. When necessary, management practices should include enhanced ecological monitoring, optimized population regulation strategies, and habitat management. Adjustments to spatial distribution patterns and targeted dietary resource management may also facilitate species coexistence (Guo et al. 2024).

In conclusion, temporal niche partitioning is recognized as a crucial driver of sympatric coexistence in ungulates. These findings provide a scientific basis for the conservation adaptive management of wild ungulate populations in Baotianman National Nature Reserve and similar temperate forest ecosystems.

## Author Contributions

**Zhaohui Xie:** conceptualization (lead), data curation (equal), formal analysis (equal), funding acquisition (equal), investigation (equal), methodology (equal), project administration (lead), resources (equal), supervision (lead), writing – original draft (equal). **Song Yao:** data curation (equal), investigation (equal), resources (equal). **Liangfu Chen:** conceptualization (equal), data curation (equal), funding acquisition (equal), investigation (equal), resources (equal). **Tongzhou Wang:** data curation (equal), investigation (equal), investigation (equal), resources (equal), resources (equal). **Tong Liu:** data curation (equal), investigation (equal), resources (equal). **Yang Zheng:** methodology (equal), validation (equal), visualization (equal). **Liwen Zhu:** data curation (supporting), formal analysis (equal), software (supporting). **Guangyi Lu:** conceptualization (equal), data curation (equal), investigation (equal), methodology (equal), software (equal), visualization (equal).

## Funding

This study was supported by the Project of Research on the Habitat of Forest Musk Deer in Baotianman National Nature Reserve, Neixiang, Henan Province (K‐H2024192, K‐H2024102).

## Conflicts of Interest

The authors declare no conflicts of interest.

## Supporting information


**Data S1:** Supporting information.

**Data S2:** Supporting information.

## Data Availability

The data that support the findings of this study are available within the article and its Supporting Information.
