# Supplementary material for: Temporal Activity and Co‐Occurrence Patterns of Sympatric Wild Ungulates in Baotianman, China
Source: Ecol Evol. 2026 Jun 30;16(7):e73897. doi: 10.1002/ece3.73897 (PMC13316962; doi:10.1002/ece3.73897)
Supplement: Supplementary file 1 — Data S1: Supporting information. [file ECE3-16-e73897-s001.docx]

**R code of Relative richness about artiodactyls in Bao Tianman**

# read CSV documents

data <-read.csv("3.csv")

setwd("C:/Users/86176/Desktop")

data<-read.xlsx("3.xlsx",sheet = 1)

# Delete blank lines

# Delete the last row and the last column

data <-data[-nrow(data), -ncol(data)]

# Convert the Photo.Time column to time format

data$Photo.Time <- as.POSIXct(data$Photo.Time, format = "%H:%M:%S")

# Count the number of hours of shooting time

data$Hour <- as.numeric(format(data$Photo.Time, "%H"))

# Define the time frame for nocturnal activity

night_hours <- c(18, 19, 20, 21, 22, 23, 0, 1, 2, 3, 4, 5, 6)

# Create a new column to mark whether or not it is nocturnal activity

data$Night_Activity <- ifelse(data$Hour %in% night_hours, "Yes", "No")

# The number of nocturnal and non-nocturnal activities is counted

night_activity_counts <- table(data$Night_Activity, data$Number.of.Animals)

# Print the results

print(night_activity_counts)

##Load the package

library(ggplot2)

# Draw a histogram of the number of nocturnal and non-nocturnal activities and save as a PDF file

g <- ggplot(data, aes(x = Night_Activity, fill = Night_Activity)) +

geom_bar() +

geom_text(stat = 'count', aes(label = ..count..), vjust = -0.5) +

labs(x = "Night Activity", y = "Count", title = "Night Activity vs. Non-night Activity") +

theme_minimal()

#ggsave("night_activity_plot.pdf", plot = g, device = "pdf")

# The proportion of nocturnal activity is calculated based on Species_cn and Night_Activity

# Count the number of different animals

unique_species <- unique(data$Species_cn)

# Create an empty data frame to store the percentage of nocturnal activity for each animal

animal_activity <- data.frame(Species_cn = character(), Number.of.Animals = numeric())

# Create an empty data frame to store the results

animal_activity <- data.frame(Species_cn = character(), Night_Activity_Count = numeric(), Total_Count = numeric(), Proportion = character())

for (species in unique_species) {

# Get the data of the current animal

species_data <- subset(data, Species_cn == species)

# Count the number of nighttime activities and the total number

night_activity_count <- sum(species_data$Night_Activity == "Yes")

total_count <- sum(species_data$Number.of.Animals)

# Calculate the percentage of nocturnal activity and convert the format to percentages

proportion <- night_activity_count / total_count * 100

proportion_formatted <- paste0(format(proportion, digits = 2), "%") # 格式化为百分比

# Add the results to the data frame

animal_activity <- rbind(animal_activity, data.frame(Species_cn = species, Night_Activity_Count = night_activity_count, Total_Count = total_count, Proportion = proportion_formatted))

}

# Set the drawing parameters, the background is pure white, and remove the coordinate axis spaces

# Draw an optimized scatter plot

# Adjust the angle and position of the abscissa label

# Draw a scatter plot and set labels and titles

# Convert Species_cn columns to characters

#Load the dplyr package

library(dplyr)

# Sort by Number.of.Animals from largest to smallest

animal_activity <- arrange(animal_activity, desc(Proportion))

# Convert Species_cn to factors and specify the order of factor levels

animal_activity$Species_cn <- factor(animal_activity$Species_cn, levels = c("西伯利亚狍", "林麝", "野猪", "小麂", "中华斑羚"))

# Remove the percent sign from the Proportion column and convert it to a numeric value

animal_activity$Proportion_numeric <- as.numeric(sub("%", "", animal_activity$Proportion))

animal_activity$Species_cn <- factor(animal_activity$Species_cn)

#Draw a scatter plot

# Converts Species_cn column to a factor, and specifies the order of the factors' levels

animal_activity$Species_cn <- factor(animal_activity$Species_cn, levels = animal_activity$Species_cn)

# Draw a scatter plot

g <- ggplot(animal_activity, aes(x = Species_cn, y = Proportion_numeric)) +

geom_point() +

labs(x = "动物名", y = "夜间活动所占比例（NRNI%）") +

scale_y_continuous(labels = scales::percent_format(scale = 1), name = "夜间活动所占比例（NRNI%）") +

theme_minimal() +

theme(panel.grid = element_blank(),

axis.text.x = element_text(angle = 45, hjust = 1),

axis.title.x = element_text(size = 12, face = "bold"),

axis.title.y = element_text(size = 12, face = "bold"),

axis.line = element_line(color = "black"), # Add axes

axis.ticks = element_line(color = "black")) # Add tick marks

ggsave("3夜行.pdf", plot = g)

###################################################################

#Seasonal Analysis

#######################################################################

# Mapping the temporal distribution of the population species

# Calculate the total number of Night_Activity and Number.of.Animals

data_summary <- data %>%

group_by(Hour) %>%

summarise(Total_Animals = sum(Number.of.Animals),

Total_Night_Activity = sum(ifelse(Night_Activity == "Yes", Number.of.Animals, 0)))

# Mapping the temporal distribution of the population species

ggplot(data_summary, aes(x = Hour, y = Total_Animals, fill = "Total Animals")) +

geom_bar(stat = "identity", position = "dodge") +

geom_bar(aes(y = Total_Night_Activity, fill = "Night Activity"), stat = "identity", position = "dodge") +

labs(x = "Hour", y = "Count", title = "Activity Time Distribution by Hour") +

scale_fill_manual(values = c("Total Animals" = "blue", "Night Activity" = "red")) +

theme_minimal()

# Convert Photo.Time to a time period

# Extraction Time Section

data$Time <- format(data$Photo.Time, "%H:%M:%S")

# Create a time period label

time_labels <- c("00:00-01:00", "01:00-02:00", "02:00-03:00", "03:00-04:00",

"04:00-05:00", "05:00-06:00", "06:00-07:00", "07:00-08:00",

"08:00-09:00", "09:00-10:00", "10:00-11:00", "11:00-12:00",

"12:00-13:00", "13:00-14:00", "14:00-15:00", "15:00-16:00",

"16:00-17:00", "17:00-18:00", "18:00-19:00", "19:00-20:00",

"20:00-21:00", "21:00-22:00", "22:00-23:00", "23:00-00:00")

# Convert time to time period labels

data$Time_Period <- factor(cut(data$Photo.Time, breaks = "1 hour"), labels = time_labels)

# Draw charts

ggplot(data, aes(x = Time_Period, fill = Night_Activity)) +

geom_bar() +

labs(x = "Time Period", y = "Count", title = "Activity Time Distribution by Time Period") +

theme_minimal() +

coord_flip()

ggplot(data, aes(x = Time_Period, fill = Night_Activity)) +

geom_bar() +

labs(x = "Time Period", y = "Count", title = "Activity Time Distribution by Time Period") +

theme_minimal() +

theme(axis.text.x = element_text(angle = 90, vjust = 0.5, hjust=1))

library(dplyr)

library(ggplot2)

# Let's assume that your data frame is data and contains columns for Species, ##Time_Period, and Number.of.Animals
# Calculate the number of each animal in each time period

data_summary1 <- data %>%group_by(Species_cn, Time_Period) %>%

summarise(Count = sum(Number.of.Animals), .groups = "drop")

# Suppose you have calculated the number of each animal for each time period and saved ##the results in the data_summary1

# Draw a line chart

ggplot(data_summary1, aes(x = Time_Period, y = Count, group = Species_cn, color = Species_cn, shape = Species_cn)) +

geom_line() +

geom_point(size = 3) +

labs(x = "Time Period", y = "Count", title = "Relative Abundance of Animals by Time Period") +

theme_minimal() +

theme(axis.text.x = element_text(angle = 90, vjust = 0.5, hjust = 1)) +

scale_shape_manual(values = 1:length(unique(data_summary1$Species_cn)))

ggplot(data_summary1, aes(x = Time_Period, y = Count, group = Species_cn, color = Species_cn, shape = Species_cn)) +

geom_line() +

geom_point(size = 3) +

labs(x = "Time Period", y = "Count", title = "Relative Abundance of Animals by Time Period") +

theme_minimal() +

theme(axis.text.x = element_text(angle = 90, vjust = 0.5, hjust = 1),

panel.grid = element_blank()) +

scale_shape_manual(values = 1:length(unique(data_summary1$Species_cn)))

ggplot(data_summary1, aes(x = Time_Period, y = Count, group = Species_cn, color = Species_cn, shape = Species_cn)) +

geom_line() +

geom_point(size = 3) +

labs(x = "Time Period", y = "Count", title = "Relative Abundance of Animals by Time Period") +

theme_minimal() +

theme(panel.grid.major = element_blank(),

panel.grid.minor = element_blank(),

axis.line = element_line(color = "black"),

axis.text.x = element_text(angle = 90, vjust = 0.5, hjust = 1),

axis.title = element_text(color = "black")) +

scale_shape_manual(values = 1:length(unique(data_summary1$Species_cn)))

# Set the shape of different animals

ggplot(data_summary1, aes(x = Time_Period, y = Count, group = Species_cn, color = Species_cn, shape = Species_cn)) +

geom_line() +

geom_point(size = 3) +

labs(x = "Time Period", y = "相对丰富度RAI%", title = "Relative Abundance of Animals by Time Period") +

theme_minimal() +

theme(axis.text.x = element_text(angle = 45, vjust = 1, hjust = 1)) +

# Rotate the x-axis text

scale_shape_manual(values = 1:length(unique(data_summary1$Species_cn)))

# Set the shape of different animals

ggplot(data_summary1, aes(x = Time_Period, y = Count, group = Species_cn, color = Species_cn, shape = Species_cn)) +

geom_line() +

geom_point(size = 3) +

labs(x = "Time Period", y = "相对丰富度RAI%", title = "Relative Abundance of Animals by Time Period") +

theme(axis.text.x = element_text(angle = 45, vjust = 1, hjust = 1),

# Set the angle and position of the text on the x-axis

axis.line = element_line(color = "black"), # Set the coordinate axis color panel.grid.major = element_blank(), # Remove the background grid

panel.grid.minor = element_blank()) + # Remove the background grid scale_shape_manual(values = 1:length(unique(data_summary1$Species_cn)))

# Set the shape of different animals
